# Empagliflozin attenuates acute kidney injury after myocardial infarction in diabetic rats

**DOI:** 10.1038/s41598-020-64380-y

**Published:** 2020-04-29

**Authors:** Atsushi Kuno, Yukishige Kimura, Masashi Mizuno, Hiroto Oshima, Tatsuya Sato, Norihito Moniwa, Marenao Tanaka, Toshiyuki Yano, Masaya Tanno, Takayuki Miki, Tetsuji Miura

**Affiliations:** 1 Department of Cardiovascular, Renal and Metabolic Medicine, Sapporo Medical University School of Medicine, Sapporo, Japan; 2 Department of Pharmacology, Sapporo Medical University School of Medicine, Sapporo, Japan; 3 Department of Cellular Physiology and Signal Transduction, Sapporo Medical University School of Medicine, Sapporo, Japan

**Keywords:** Mechanisms of disease, Acute kidney injury

## Abstract

Acute kidney injury (AKI) predicts poor prognosis in patients with acute myocardial infarction (MI) and diabetes mellitus (DM) is an independent risk factor of AKI. Recent clinical studies have shown the beneficial effects of sodium-glucose cotransporter 2 (SGLT2) inhibitors on cardiovascular and renal outcomes in patients with DM. We recently reported that canagliflozin normalized susceptibility of diabetic rats to AKI after acute MI via β-hydroxybutyrate-mediated suppression of NOX expression. Here we examined whether the same renoprotective effect is shared by empagliflozin. Serum creatinine levels were not changed by MI induced by coronary artery occlusion in LETO, non-diabetic control rats, and OLETF, obese type 2 diabetic rats. However, immunohistochemistry revealed that MI increased renal expression of NGAL and KIM-1, early markers of tubular injury, by 3.2-fold and 2.6-fold, respectively, in OLETF. These increases in injury markers were not observed in LETO. Pretreatment with empagliflozin of OLETF for 2 weeks improved hyperglycemia, increased blood β-hydroxybutyrate level, and suppressed MI-induced expression of NGAL and KIM-1. Empagliflozin suppressed upregulation of NOX2 and NOX4 in the kidney of OLETF. Taken together with the results of our previous study, it was concluded that treatment with the SGLT2 inhibitor protects the diabetic kidney from MI-induced AKI.

## Introduction

Acute kidney injury (AKI) is associated with poor prognosis of patients with acute myocardial infarction (MI)^1,2^. It is well known that diabetes mellitus (DM) is an independent risk factor of AKI^1,2^. We previously found that Otsuka Long-Evans Tokushima Fatty rats (OLETF), a model of type 2 DM, showed elevations in AKI markers such as neutrophil gelatinase-associated lipocalin (NGAL) and kidney injury molecule-1 (KIM-1) in the kidney after MI without an increase in serum creatinine (sCr) level^3,4^. This type of renal injury is consistent with subclinical AKI, defined as a condition in which there is an elevation in AKI markers without an increase in sCr and/or a reduction in urine output in a clinical setting^5,6^. Importantly, the increase in the AKI marker predicts a greater risk of adverse outcomes even without an increase in sCr in critically ill patients^5,6^. Using the diabetic rat model, we identified enhanced activation of renal toll-like receptor (TLR) and increased renal oxidative stress as mechanisms by which DM increases susceptibility to AKI after MI^3,4^. However, it has been unknown whether hypoglycemic drugs attenuate AKI in diabetes.

Recent clinical studies have shown the beneficial effects of sodium-glucose cotransporter 2 (SGLT2) inhibitors on renal outcomes in patients with DM^7–9^. In addition, these clinical trials suggest that SGLT2 inhibitors may prevent AKI in diabetic patients although different effects of SGLT2 inhibitors on AKI are pointed out^10^. We recently found that canagliflozin, the SGLT2 inhibitor, normalizes susceptibility to AKI after MI by reduction in renal oxidative stress via elevated β-hydroxybutyrate (βOHB) in OLETF^4^. However, it remains unknown whether such a renoprotection is provided by other SGLT2 inhibitors in a similar manner. In this study, therefore, we examined whether empagliflozin also attenuates MI-induced AKI in OLETF.

## Results

**Figure 1:**
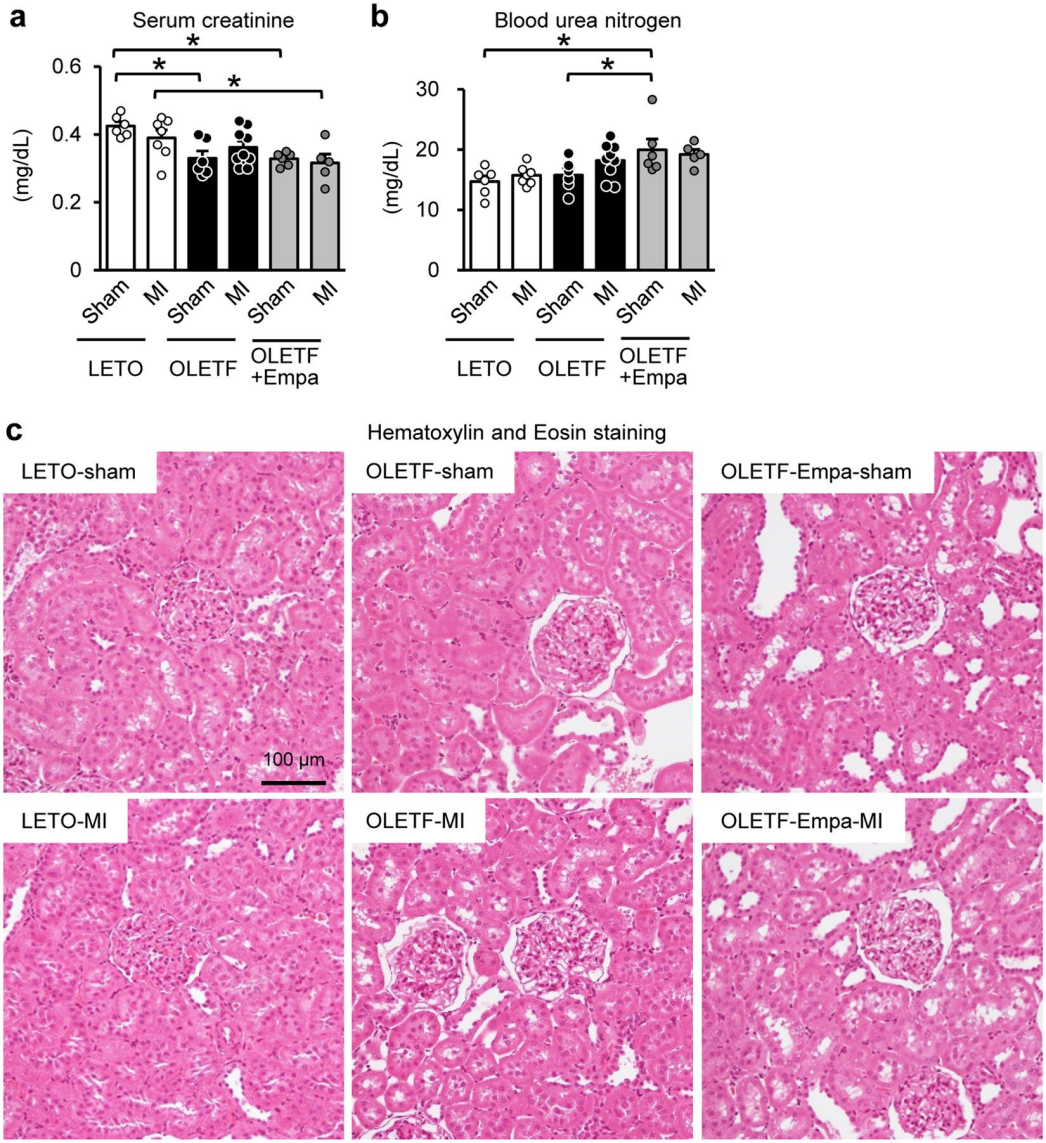
Renal parameters after surgery in LETO and OLETF. Levels of serum creatinine (**a**) and blood urea nitrogen (**b**) at 12 h after sham operation or coronary artery occlusion to induce myocardial infarction (MI) in LETO, OLETF, and empagliflozin (Empa)-treated OLETF (N = 5–9). (**c**) Representative images of Hematoxylin and eosin staining. *P < 0.05.

In this study, we analyzed blood and kidney samples obtained in our previous study^11,12^. OLETF and Long-Evans Tokushima Otsuka rats (LETO), non-diabetic control, at ages of 25-30 weeks were pretreated with a vehicle or empagliflozin (10 mg/kg/day) for 2 weeks before surgery. After fasting for 12 h, blood glucose and βOHB levels were measured and rats underwent coronary artery ligation or a sham operation. Kidney tissues and blood were sampled at 12 h after surgery, because the mortality rate at 24–48 h after MI was high in OLETF^11,13^. Blood glucose level before surgery (i.e., 12 h after fasting) was significantly higher in vehicle-treated OLETF (165 ± 9 mg/dL, N = 20) than that in LETO (121 ± 3 mg/dL, N = 20), and empagliflozin significantly reduced the level in OLETF (117 ± 7 mg/dL, N = 20) as previously reported^11,12^. Blood βOHB levels were comparable in LETO (0.77 ± 0.04 mM, N = 20) and OLETF (0.62 ± 0.03 mM, N = 20) before surgery but were significantly increased in empagliflozin-treated OLETF (1.20 ± 0.09 mM, N = 20) as is the case with canagliflozin-treated OLETF^4^. Among sham-operated rats, sCr level was lower in OLETF than in LETO (Fig. 1a), reflecting glomerular hyperfiltration associated with diabetes in this model^3,4,14^. Neither MI nor empagliflozin changed the sCr level in LETO and OLETF. Blood urea nitrogen (BUN) levels after surgery were also similar in sham-operated LETO and OLETF and were not changed by MI (Fig. 1b). The BUN level was significantly higher in empagliflozin-treated OLETF than LETO and OLETF. However, MI did not change BUN levels in empagliflozin-treated OLETF. There were no significant differences in blood levels of Na^+^, Cl^−^, K^+^, and Ca^2+^ among groups (Table 1). Although clinical studies have shown an increase in blood hematocrit level by SGLT2 inhibitors^15,16^, empagliflozin treatment did not increase hematocrit level in OLETF (Table 1).

**Table 1:** Electrolyte and hematocrit levels in rats after surgery.

	LETO		OLETF		OLETF + EMPA	
	Sham	MI	Sham	MI	Sham	MI
Na^+^ (mM)	138.4 ± 0.7	138.2 ± 0.7	137.9 ± 0.4	136 ± 0.8	139.5 ± 0.9	137.5 ± 1.2
Cl^−^ (mM)	106.8 ± 0.5	106.1 ± 0.7	107.2 ± 0.4	106.4 ± 0.7	108.2 ± 0.8	107.1 ± 0.8
K^+^ (mM)	3.69 ± 0.10	3.55 ± 0.08	3.67 ± 0.08	3.86 ± 0.10	3.50 ± 0.08	3.60 ± 0.09
Ca^2+^ (mM)	1.09 ± 0.02	1.07 ± 0.03	1.11 ± 0.02	1.15 ± 0.03	1.11 ± 0.03	1.09 ± 0.04
Hct (%)	42.8 ± 0.8	45.3 ± 0.6^*^	44.1 ± 0.5	44.5 ± 0.6	39.3 ± 0.9^*‡^	40.4 ± 0.8^†§^

Values are mean ± SEM. N = 10.

EMPA, empagliflozin; MI, myocardial infarction; Hct, hematocrit.

*p < 0.05 vs. LETO-sham.

^†^p < 0.05 vs. LETO-MI.

^‡^p < 0.05 vs. OLETF-sham.

^§^p < 0.05 vs. OLETF-MI.

**Figure 2:**
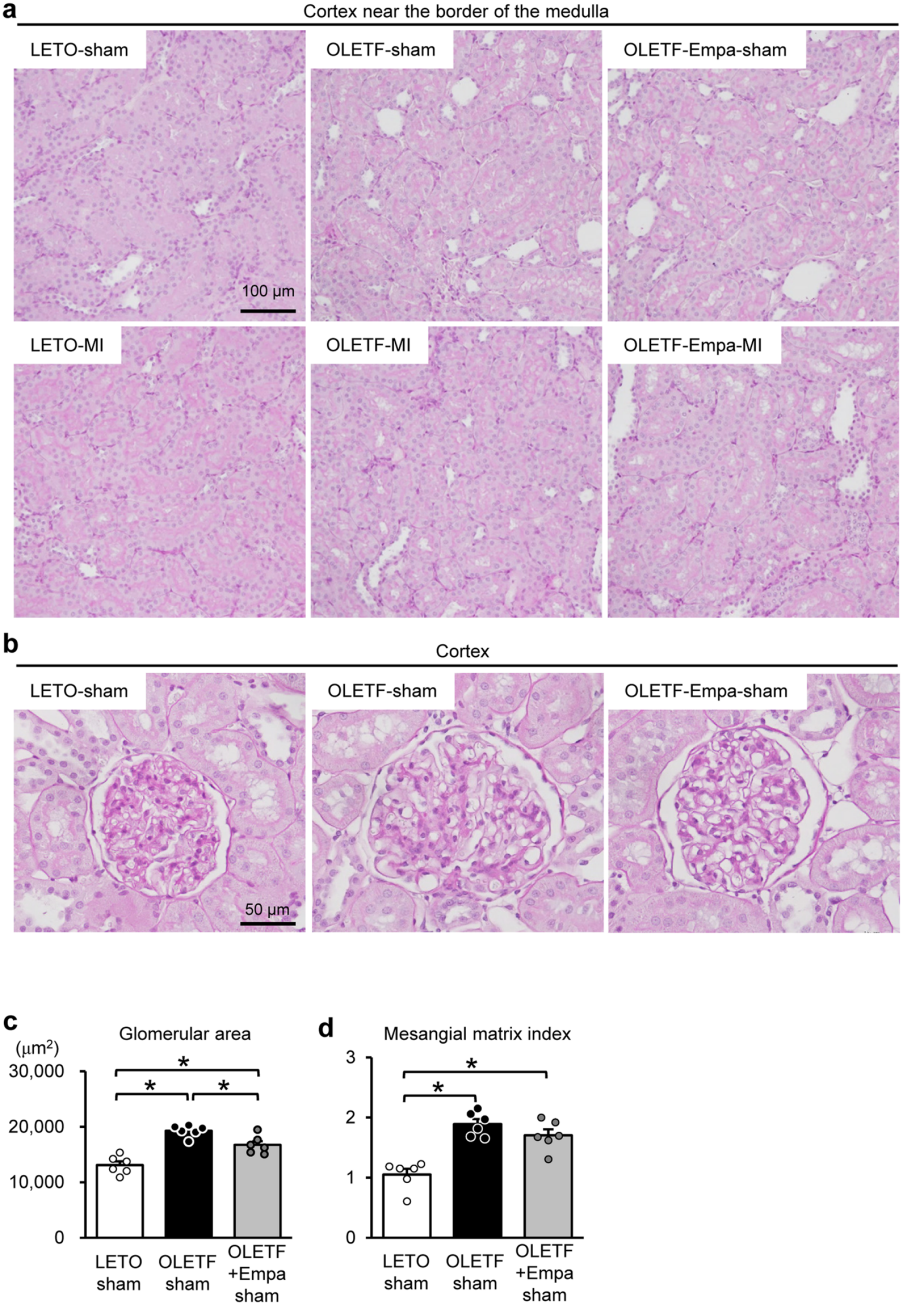
Effects of diabetes and empagliflozin on tubular cells and glomeruli. Representative images of tubules in the cortex near the border of the medulla (**a**) and glomeruli in the cortex (**b**) assessed by PAS staining in the kidney sections from LETO, OLETF, and empagliflozin (Empa)-treated OLETF. Summary data of glomerular areas (**c**) and mesangial matrix index in glomerulus (**d**) in sham-operated rats. N = 6. *P < 0.05.

**Figure 3:**
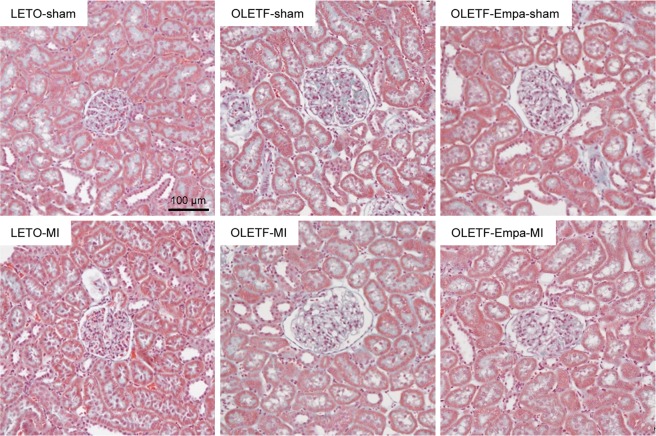
Masson’s Trichrome staining in the kidney. Representative images of Masson’s Trichrome staining in the kidney sections sampled from LETO, OLETF, and empagliflozin (Empa)-treated OLETF.

Hematoxylin and eosin (HE) staining (Fig. 1c) and periodic acid Schiff (PAS) staining (Fig. 2a) in the kidney section did not show detectable abnormalities in tubular cells, such as loss of the brush border, tubular dilation, cast formation, and cell lysis, in LETO and OLETF regardless of MI as shown in our previous report^3,4^. Effect of empagliflozin on tubular cells was not detected in histology stained with HE and PAS (Figs. 1c and 2a). Among sham-operated rats, glomerular area was significantly larger in OLETF than that in LETO (Fig. 2b,c) as we previously reported^17^. Empagliflozin treatment for two weeks significantly reduced glomerular area in OLETF (Fig. 2b,c). Mesangial matrix level in the glomerulus determined with mesangial matrix index was significantly higher in OLETF than that LETO (Fig. 2b,d). Empagliflozin modestly decreased the index in OLETF; however, the difference did not reach statistical significance. Masson’s Trichrome staining demonstrated that there was no interstitial fibrosis in LETO and OLETF, regardless of Ml and empagliflozin treatment (Fig. 3).

**Figure 4:**
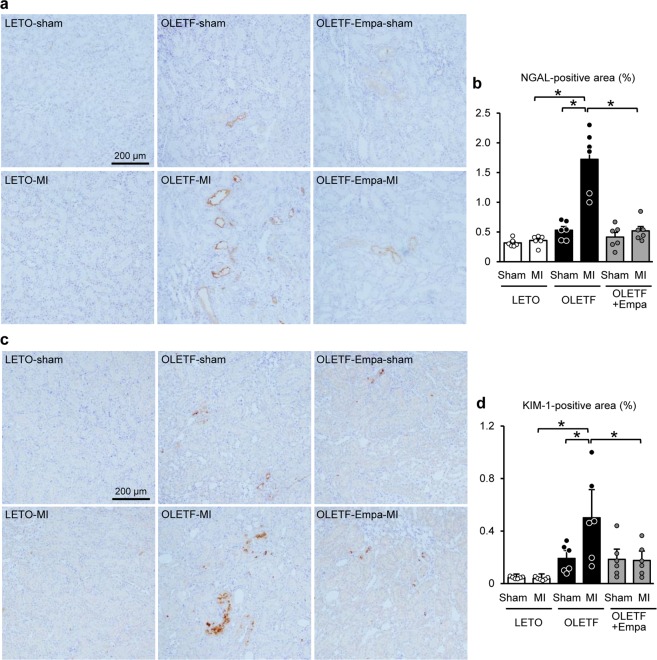
Immunohistochemistry for NGAL and KIM-1 in the kidney. Representative images of immunohistochemistry for NGAL (**a**) and KIM-1 (**c**) in kidney sections from LETO, OLETF, and OLETF treated with empagliflozin (Empa) that were sampled after a sham operation (Sham) or myocardial infarction (MI). Summary data of NGAL- (**b**) and KIM-1- (**d**) positive areas. N = 6. *P < 0.05.

We next analyzed expression levels of NGAL and KIM-1 in the kidney as early markers of tubular injury^3,4^. Immunohistochemistry showed no differences in NGAL- and KIM-1-positive areas among sham-operated rats (Fig. 4a–d). MI increased NGAL- and KIM-1-positive areas detected in tubular cells in OLETF but not in LETO, and these increases were significantly suppressed by empagliflozin (Fig. 4a–d).

**Figure 5:**
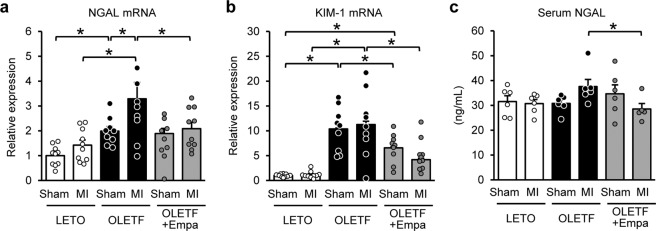
Levels of NGAL and KIM-1 mRNA in the kidney and serum NGAL levels after surgery. Renal mRNA levels of NGAL (**a**), KIM-1 (**b**). N = 9–10. (**c**) Serum NGAL levels 12 h after surgery. N = 5–6. *P < 0.05.

Renal mRNA levels of NGAL and KIM-1 were significantly higher in OLETF than in LETO regardless of MI induction (Fig. 5a,b). MI elevated NGAL mRNA level in OLETF, which was blocked by empagliflozin (Fig. 5a). Empagliflozin reduced KIM-1 mRNA levels in OLETF with or without MI (Fig. 5b). Serum NGAL levels were comparable among sham-operated LETO, OLETF, and empagliflozin-treated OLETF (Fig. 5c). Serum NGAL level was not changed by MI in LETO, whereas MI tended to increase the level in OLETF (P = 0.056). Empagliflozin treatment significantly reduced NGAL level in the serum in OLETF after MI (Fig. 5c).

**Figure 6:**
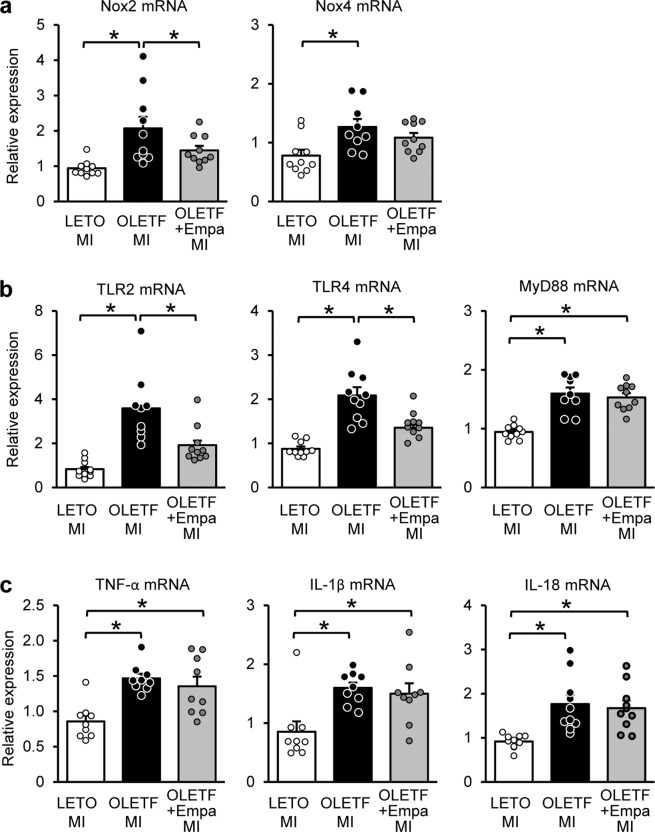
Renal mRNA levels of NOXs, TLRs, and inflammatory cytokines in the kidney. Renal mRNA levels of (**a**) NOX2 and NOX4, (**b**) TLR2, TLR4, and MyD88, and (**c**) TNF-α, IL-1β, and IL-18 in LETO, OLETF, and empagliflozin (Empa)-treated OLETF after myocardial infarction (MI). N = 9–10 in each group. *P < 0.05.

Finally, we examined how empagliflozin attenuated AKI in OLETF. We recently reported that canagliflozin suppressed upregulation of NADPH oxidase 2 (NOX2) and NOX4, which mediate renal injury^18,19^, via an increased βOHB in OLETF^4^. Renal mRNA levels of NOX2 were upregulated in OLETF after MI, which was significantly suppressed by empagliflozin (Fig. 6a). In OLETF after MI, NOX4 mRNA levels were also upregulated; however, such upregulation was not observed in empagliflozin-treated OLETF (Fig. 6a).

We previously found that increased expression of TLRs in the kidney causes inflammation, leading to AKI after MI in OLETF^3^. Renal mRNA levels of TLR2, TLR4, and a TLR adaptor protein MyD88 were significantly higher in MI-operated OLETF than those in MI-operated LETO (Fig. 6b). Renal mRNA levels of tumor necrosis factor (TNF)-α, interleukin-1β (IL-1β), and IL-18 were significantly higher in OLETF with MI than those in LETO (Fig. 6c). Empagliflozin treatment significantly suppressed upregulation of TLR2 and TLR4 (Fig. 6b) in OLETF while mRNA levels of MyD88, TNF-α, IL-1β, and IL-18 were not changed by empagliflozin (Fig. 6b and c).

## Discussion

Pretreatment with empagliflozin attenuated AKI, defined by elevation of renal NGAL and KIM-1 levels, after MI in OLETF in a manner similar to that of pretreatment with canagliflozin^4^. MI did not change either sCr or the BUN level in OLETF. As discussed in our previous reports^3,4^, this type of renal injury is consistent with subclinical AKI^5,6^. Importantly, recent clinical data have shown that even subclinical AKI is associated with adverse outcomes^5,6^. Although it remains unclear whether an intervention that prevents subclinical AKI improves the outcome of patients, protection against subclinical AKI by empagliflozin may have a clinical impact in patients with type 2 DM.

Inhibition of SGLT2 blocks reabsorption of glucose as well as sodium in the proximal tubules, which increases sodium concentration at the macula densa, leading to normalization of tubuloglomerular feedback and attenuation of renal hyperfiltration^20^. Empagliflozin treatment for 2 weeks significantly attenuated enlargement of the glomerular area in OLETF (Fig. 2b,c). A previous study also showed that treatment of Akita mice, a model of type 1 diabetes, with empagliflozin for 15 weeks reduced blood glucose level and normalized glomerular size, which was associated with the reductions in glomerular filtration rate^21^. Therefore, the protection afforded by empagliflozin observed in OLETF could result from attenuation of hyperfiltration. It is noteworthy that relatively short duration of treatment for 2 weeks was sufficient to the histological improvement in the glomerular size. On the other hand, the reduction in mesangial matrix level by empagliflozin did not reach a statistical significance, suggesting that longer treatment is necessary to attenuate mesangial expansion.

Elevations in renal mRNA levels of NOXs were attenuated by empagliflozin (Fig. 6a). We recently reported that renoprotection by canagliflozin was associated with reductions in oxidative stress and expression levels of NOXs in the kidney and that blood βOHB levels before MI were inversely correlated with renal NGAL levels in OLETF^4^. In addition, β-hydroxybutyrate was shown to repress NOX expression in renal tubular cells^4^ as well as PC12 cells^22^. We found a similar elevation in blood βOHB level caused by empagliflozin before induction of MI in OLETF^11^. Therefore, suppression of NOX upregulation via βOHB possibly underlies the effect of empagliflozin. Additionally, empagliflozin affords cardioprotection in OLETF. We recently showed that empagliflozin preserved ATP and reduced oxidative stress in the heart after MI in OLETF^11^. Thus, attenuation of heart failure might have contributed to the renoprotection by empagliflozin. On the other hand, it is unlikely that empagliflozin treatment attenuated inflammation, a possible mechanism of AKI after MI in diabetes^3^, because the increases in renal mRNA levels of TNF-α, IL-1β, and IL-18 in OLETF were not suppressed by empagliflozin (Fig. 6c) as was found in our previous study using canagliflozin^4^. Therefore, the present findings support the notion that the mechanisms by which canagliflozin and empagliflozin prevent AKI after MI in DM may be similar.

In this study, empagliflozin treatment significantly increased BUN level in OLETF (Fig. 1b). In consistent with this finding, some clinical studies have shown that the SGLT2 inhibitor resulted in mild elevation in BUN level in patients with type 2 DM^23,24^. In our previous study^4^, elevation in BUN was also observed in canagliflozin-treated OLETF without a change in sCr level. In the present study, the sCr level was not increased by empagliflozin (Fig. 1a), suggesting BUN elevation independent of the change in glomerular filtration. Because hematocrit level was not increased by empagliflozin (Table 1), dehydration is unlikely to be a cause of the increase in BUN. In the present study, rats were subjected to fasting for 24 h before blood sampling. We previously observed that BUN elevation by canagliflozin was modest after 12 h of fasting and that longer fasting period of 24 h was required for significant BUN elevation by canagliflozin^4^. In addition, it is reported that administration of dapagliflozin, another SGLT2 inhibitor, enhances lipid oxidation as well as protein catabolism^25^. Therefore, it is possible that longer fasting with loss of glucose by SGLT2 inhibition may have caused a more severe starved condition, leading to enhancement of protein catabolism and therefore BUN elevation.

It is reported that administration of the dipeptidyl peptidase 4 (DPP4) inhibitor, another glucose lowering drug, is associated with reduced risk of AKI in patients with DM^26^. In a non-diabetic rat model, treatment with the DPP4 inhibitor sitagliptin attenuated tubular injury after renal ischemia reperfusion^27^. It remains unclear whether combination of the DPP4 inhibitor with the SGLT2 inhibitor provide more beneficial effects against AKI after MI in OLETF. Unlike in the case of the SGLT2 inhibitor, the DPP4 inhibitor has little effect on ketone body production. Although TLR-mediated inflammation is involved in AKI after MI in DM^3^, plasma and renal cytokine levels were not reduced by sitagliptin^27^. Therefore, it is unlikely that the DPP4 inhibitor provides additive renoprotection in diabetic rats treated with the SGLT2 inhibitor after MI. However, we cannot exclude the possibility that it is meaningful to combine both approach in case of more severe AKI including a model of renal ischemia reperfusion.

There are several limitations in this study. First, we could not measure urinary level of NGAL or KIM-1 because of a shortage of urine samples. However, it has been reported that there was a strong correlation between levels of urinary NGAL and renal NGAL mRNA in lipopolysaccharide-treated rats^28^. In addition, urinary KIM-1 levels were reported to be closely correlated with tissue KIM-1 expression in renal biopsy samples from various renal diseases^29^. These findings suggest that tissue levels of these markers reflect their urinary levels in this study. Second, we could not evaluate the long-term effects of empagliflozin on kidney injury after MI in OLETF because the mortality rates at 24 and 48 h after MI are very high in OLETF^11,13^. To address this issue, the establishment of an AKI model that has a minimum effect on mortality in the acute phase in OLETF is needed. Third, it remains unclear whether empagliflozin also normalizes diabetes-induced susceptibility to AKI by other etiologies, including renal ischemia-reperfusion, contrast medium, and cisplatin. Further studies are needed to clarify this issue.

In conclusion, empagliflozin provides protection against subclinical AKI after MI in DM in a manner similar to that of canagliflozin. Such a renoprotective effect may contribute to improvements in clinical outcomes by SGLT2 inhibitors in patients with DM.

## Methods

This study was conducted according to The Animal Guideline of Sapporo Medical University and approved by the Animal Use Committee of Sapporo Medical University.

We used male OLETF and LETO rats at ages of 25–30 weeks. LETO and OLETF were purchased from Sankyo Labo Service Corporation (Tokyo, Japan). Empagliflozin (10 mg/kg/day) and the vehicle (dimethyl sulfoxide and polyethylene glycol; 1:1 vol:vol) were administered subcutaneously via osmotic minipumps (Alzet, Cupertino, CA, USA) for two weeks before surgery. We selected this dose of empagliflozin based on a previous study using obese rats^30^ and our preliminary study showing that subcutaneous administration of empagliflozin at 10 mg/kg/day for 2 weeks was sufficient to reduce blood glucose level and to increase urine glucose level in OLETF (data not shown). Empagliflozin was provided by Boehringer Ingelheim (Ingelheim am Rhein, Germany). After fasting for 12 h, rats were anesthetized with isoflurane inhalation and then were intubated and ventilated with a rodent respirator (model 683, Harvard Apparatus, South Natick, MA). Blood glucose and βOHB were measured using a Glutest-mint (Sanwa Kagaku Kenkyusho, Nagoya, Japan) and Presicion Xceed (Abbot, Chicago, IL), respectively. The heart was exposed via left thoracotomy, and then a marginal branch of the left coronary artery was permanently ligated using a 5-0 silk thread to induce MI. The surgical wounds were repaired, and the rats were returned to their cages. All rats were allowed ad-lib access to water but were restricted from food for 12 h. The rats were re-anesthetized and ventilated to sample kidney tissues and blood at 12 h after MI. The abdomen was opened, and the kidneys were excised immediately after occluding renal arteries and veins for immersing in iced-cold saline. One kidney was quickly frozen in liquid nitrogen and stored at −80 °C until use for biochemical analyses, and the other kidney was fixed with 10% formaldehyde for histology.

Glomerular area and mesangial matrix level were analyzed in images from PAS staining. At least 35 glomeruli were randomly selected from five images in each kidney section. Glomerular areas were determined by using ImageJ software. Mesangial matrix level was scored semiquantitatively, and relative size of PAS-positive area of glomerulus was rated as follows: 0, 0%; 1, 1–25%; 2, 26–50%; 3, 51–75%; and 4, >75%. The average of the score was presented as a mesangial matrix index.

Immunohistochemistry for NGAL and KIM-1 were analyzed as previously reported^3,4^. Briefly, formaldehyde-fixed paraffin sections (3 μm) were stained using primary antibodies against KIM-1 (R&D Systems, AF3689, 1:20) and NGAL (Santa Cruz Biotechnology, sc-50531, 1:100). NGAL- and KIM-1-positive areas were determined in 10 randomly selected fields from six kidneys in each group.

Levels of NGAL in serum samples obtained at 12 h after surgery were determined with a commercial ELISA kit (KIT046) from BioPorto Diagnostics according to the protocol provided by the manufacturer.

Total RNA was isolated from frozen kidney tissues by using an RNeasy Fibrous Tissue Mini Kit (Qiagen, Valencia, CA, USA). First-strand cDNA was synthesized using a SuperScript VILO™ cDNA Synthesis Kit (Life Technologies). DNA amplification was performed in ABI PRISM7500 (Life Technologies) by using Taqman Universal PCR Master Mix (Applied Biosystems, Inc). Taqman gene expression assays used in this study were as follows: KIM-1 (Rn00597703_m1), NGAL (Rn00590612_m1), NOX2 (Rn00576710_m1), NOX4 (Rn00585380_m1), TLR2 (Rn02133647_s1), TLR4 (Rn00569848_m1), MyD88 (Rn01640049_m1), TNF-α (Rn99999017_m1), IL-1β (Rn00580432_m1), IL-18 (Rn01422083_m1), and β-actin. (Rn00667869_m1). All assays were performed in duplicate and by the standard curve method using serial cDNA dilution. β-actin served as an internal control.

Data are presented as means ± SEM. Differences between groups were assessed by one-way or two-way analysis of variance. The Student-Newman-Keuls test was used for multiple comparisons. P < 0.05 was considered statistically significant.

## Data Availability

All datasets generated during the current study are available from the corresponding author upon reasonable request.

## References

[1] Goldberg A, Kogan E, Hammerman H, Markiewicz W, Aronson D (2009). The impact of transient and persistent acute kidney injury on long-term outcomes after acute myocardial infarction. Kidney Int..

[2] Fox CS (2012). Short-term outcomes of acute myocardial infarction in patients with acute kidney injury: a report from the national cardiovascular data registry. Circulation.

[3] Ohno K (2017). Diabetes increases the susceptibility to acute kidney injury after myocardial infarction through augmented activation of renal Toll-like receptors in rats. Am. J. Physiol. Heart Circ. Physiol.

[4] Kimura Y (2019). Canagliflozin, a sodium-glucose cotransporter 2 inhibitor, normalizes renal susceptibility to type 1 cardiorenal syndrome through reduction of renal oxidative stress in diabetic rats. J. Diabetes Investig..

[5] Haase M (2011). The outcome of neutrophil gelatinase-associated lipocalin-positive subclinical acute kidney injury: a multicenter pooled analysis of prospective studies. J. Am. Coll. Cardiol..

[6] Albert C (2018). Urinary biomarkers may provide prognostic information for subclinical acute kidney injury after cardiac surgery. J. Thorac. Cardiovasc. Surg..

[7] Wanner C (2016). Empagliflozin and Progression of Kidney Disease in Type 2 Diabetes. N. Engl. J. Med..

[8] Perkovic V (2019). Canagliflozin and Renal Outcomes in Type 2 Diabetes and Nephropathy. N. Engl. J. Med..

[9] Wiviott SD (2019). Dapagliflozin and Cardiovascular Outcomes in Type 2 Diabetes. N. Engl. J. Med..

[10] Chu C, Lu YP, Yin L, Hocher B (2019). The SGLT2 Inhibitor Empagliflozin Might Be a New Approach for the Prevention of Acute Kidney Injury. Kidney Blood Press. Res..

[11] Oshima H (2019). Empagliflozin, an SGLT2 Inhibitor, Reduced the Mortality Rate after Acute Myocardial Infarction with Modification of Cardiac Metabolomes and Antioxidants in Diabetic Rats. J. Pharmacol. Exp. Ther..

[12] Mizuno M (2018). Empagliflozin normalizes the size and number of mitochondria and prevents reduction in mitochondrial size after myocardial infarction in diabetic hearts. Physiol. Rep..

[13] Murase H (2015). Inhibition of DPP-4 reduces acute mortality after myocardial infarction with restoration of autophagic response in type 2 diabetic rats. Cardiovasc. Diabetol..

[14] Kawano K, Mori S, Hirashima T, Man ZW, Natori T (1999). Examination of the pathogenesis of diabetic nephropathy in OLETF rats. J. Vet. Med. Sci..

[15] Lambers Heerspink HJ, de Zeeuw D, Wie L, Leslie B, List J (2013). Dapagliflozin a glucose-regulating drug with diuretic properties in subjects with type 2 diabetes. Diabetes Obes. Metab..

[16] Inzucchi SE (2018). How Does Empagliflozin Reduce Cardiovascular Mortality? Insights From a Mediation Analysis of the EMPA-REG OUTCOME Trial. Diabetes Care.

[17] Muratsubaki S (2017). Suppressed autophagic response underlies augmentation of renal ischemia/reperfusion injury by type 2 diabetes. Sci. Rep..

[18] Karim AS (2015). Nox2 is a mediator of ischemia reperfusion injury. Am. J. Transpl..

[19] Yang Q (2018). Nox4 in renal diseases: An update. Free. Radic. Biol. Med..

[20] Cherney DZ (2014). Renal hemodynamic effect of sodium-glucose cotransporter 2 inhibition in patients with type 1 diabetes mellitus. Circulation.

[21] Vallon V (2014). SGLT2 inhibitor empagliflozin reduces renal growth and albuminuria in proportion to hyperglycemia and prevents glomerular hyperfiltration in diabetic Akita mice. Am. J. Physiol. Ren. Physiol.

[22] Kong G (2017). The Ketone Metabolite beta-Hydroxybutyrate Attenuates Oxidative Stress in Spinal Cord Injury by Suppression of Class I Histone Deacetylases. J. Neurotrauma.

[23] Sha S (2014). Effect of the sodium glucose co-transporter 2 inhibitor canagliflozin on plasma volume in patients with type 2 diabetes mellitus. Diabetes Obes. Metab..

[24] Yale JF (2013). Efficacy and safety of canagliflozin in subjects with type 2 diabetes and chronic kidney disease. Diabetes Obes. Metab..

[25] Mudaliar S (2014). Changes in insulin sensitivity and insulin secretion with the sodium glucose cotransporter 2 inhibitor dapagliflozin. Diabetes Technol. Ther..

[26] Chao CT, Wang J, Wu HY, Chien KL, Hung KY (2017). Dipeptidyl peptidase 4 inhibitor use is associated with a lower risk of incident acute kidney injury in patients with diabetes. Oncotarget.

[27] Reichetzeder C (2017). Head-to-head comparison of structurally unrelated dipeptidyl peptidase 4 inhibitors in the setting of renal ischemia reperfusion injury. Br. J. Pharmacol..

[28] Han M, Li Y, Liu M, Li Y, Cong B (2012). Renal neutrophil gelatinase associated lipocalin expression in lipopolysaccharide-induced acute kidney injury in the rat. BMC Nephrol..

[29] van Timmeren MM (2007). Tubular kidney injury molecule-1 (KIM-1) in human renal disease. J. Pathol..

[30] Vickers SP (2014). Combination of the sodium-glucose cotransporter-2 inhibitor empagliflozin with orlistat or sibutramine further improves the body-weight reduction and glucose homeostasis of obese rats fed a cafeteria diet. Diabetes Metab. Syndr. Obes..

